# Physiological Basis of Noise-Induced Hearing Loss in a Tympanal Ear

**DOI:** 10.1523/JNEUROSCI.2279-19.2019

**Published:** 2020-04-08

**Authors:** Ben Warren, Georgina E. Fenton, Elizabeth Klenschi, James F.C. Windmill, Andrew S. French

**Affiliations:** ^1^Department of Neuroscience, Psychology and Behavior, University of Leicester, Leicester, LE1 7RH, United Kingdom,; ^2^Centre for Ultrasonic Engineering, Department of Electronic and Electrical Engineering, University of Strathclyde, Glasgow, G1 1XW, United Kingdom, and; ^3^Department of Physiology and Biophysics, Dalhousie University, Halifax, Nova Scotia B3H 4R2, Canada

**Keywords:** auditory neurons, auditory receptors, auditory transduction, hearing, mechanotransduction ion channel, noise-induced hearing loss

## Abstract

Acoustic overexposure, such as listening to loud music too often, results in noise-induced hearing loss. The pathologies of this prevalent sensory disorder begin within the ear at synapses of the primary auditory receptors, their postsynaptic partners and their supporting cells. The extent of noise-induced damage, however, is determined by overstimulation of primary auditory receptors, upstream of where the pathologies manifest. A systematic characterization of the electrophysiological function of the upstream primary auditory receptors is warranted to understand how noise exposure impacts on downstream targets, where the pathologies of hearing loss begin. Here, we used the experimentally-accessible locust ear (male, *Schistocerca gregaria)* to characterize a decrease in the auditory receptor's ability to respond to sound after noise exposure. Surprisingly, after noise exposure, the electrophysiological properties of the auditory receptors remain unchanged, despite a decrease in the ability to transduce sound. This auditory deficit stems from changes in a specialized receptor lymph that bathes the auditory receptors, revealing striking parallels with the mammalian auditory system.

**SIGNIFICANCE STATEMENT** Noise exposure is the largest preventable cause of hearing loss. It is the auditory receptors that bear the initial brunt of excessive acoustic stimulation, because they must convert excessive sound-induced movements into electrical signals, but remain functional afterward. Here we use the accessible ear of an invertebrate to, for the first time in any animal, characterize changes in auditory receptors after noise overexposure. We find that their decreased ability to transduce sound into electrical signals is, most probably, due to changes in supporting (scolopale) cells that maintain the ionic composition of the ear. An emerging doctrine in hearing research is that vertebrate primary auditory receptors are surprisingly robust, something that we show rings true for invertebrate ears too.

## Abstract (precis)

Acoustic overexposure, such as listening to loud music too often, results in noise-induced hearing loss. The pathologies of this prevalent sensory disorder begin within the ear at synapses of the primary auditory receptors, their postsynaptic partners and their supporting cells. The extent of noise-induced damage, however, is determined by overstimulation of primary auditory receptors, upstream of where the pathologies manifest.

## Introduction

Our senses endow us with an incredible subjective experience of our world. The gatekeepers of this external sensory environment, sensory receptors, selectively encode stimuli into electrical signals to give us our five senses. Overstimulation of sensory neurons, however, leads to damage and the sound-sensitive cells in our ears are particularly prone to permanent injury (Lundström and Johansson, 1986; Young, 1988; Salvi and Boettcher, 2008). As a result, the most common sensory impairment in humans is hearing loss and the majority of preventable hearing loss is due to excessive noise exposure.

The ears of all animals have specialized ciliated sound-sensitive receptor cells, likely of a common evolutionary origin (Fritzsch and Beisel, 2004), that transduce sound into electrical potentials. Critical to this process of auditory transduction are stretch-sensitive ion channels, embedded in the membranes of the cilia, which open in response to sound (Gillespie and Walker, 2001; Albert and Kozlov, 2016). Common to arthropod hearing is the maintenance of a specialized cation-rich lymph, by supporting cells, which bathe the cilia of auditory receptor cells. In insects, a single supporting scolopale cell encloses the cilium and pumps cations into the receptor lymph. Similarly, in the cochlea of mammals (in the stria vascularis) three groups of supporting cells pump cations into the endolymph. This generates a high electrochemical gradient such that when the transduction ion channels are opened by sound cations flow into the auditory receptor cells to generate the primary electrical signal; the receptor potential. In Orthoptera, such as locusts and crickets, the transduction potential is converted into a small dendritic spike that travels along a single dendrite of the neuron (Hill, 1983b; Oldfield and Hill, 1986) to the soma. There, most probably in the axon hillock, it triggers a larger axonal spike, at its basal end, that carries auditory information to the CNS (Warren and Matheson, 2018).

Two decades ago, it was assumed that the pathologies of hearing loss (such as spiral ganglion neuron loss) result from, and after, a loss of “upstream” hair cells (Bohne and Harding, 2000). A decade ago this doctrine was challenged as noise exposure was found to reduce hearing thresholds without any loss of hair cells (Kujawa and Liberman, 2009; Lin et al., 2011). Hair cells, therefore, appear more resilient than first thought and are not necessarily the primary source of hearing loss. None-the-less, in response to loud noise, the primary auditory receptors of any animal will experience an excessive influx of cations. This influx leads to production of reactive oxygen species by mitochondria (Yamane et al., 1995), damage of mitochondria (Christie et al., 2013) and glutamatergic excitotoxicity (Pujol et al., 1993; Kujawa and Liberman, 2009) and synaptic damage (Liberman et al., 2015), which can trigger apoptotic and necrotic pathways (Wagner and Shin, 2019). A recurring target of damage in auditory systems is in the supporting cells (Yamane et al., 1995; Yamasoba et al., 1998; Shi and Nuttall, 2003; Shi et al., 2015), which, when damaged or genetically altered, fail to pump a normal amount of cations into the receptor lymph that bathes the cilia of the primary auditory receptors (Vassout, 1984; Ma et al., 1995; Li et al., 1997; Hirose and Liberman, 2003), which can lead to auditory pathologies (Roy et al., 2013; Christie and Eberl, 2014).

Despite the severe effects of noise in auditory systems, there is no systematic understanding of changes in the electrophysiological properties and function of the auditory receptor cells themselves shortly after noise exposure for any animal. The locust ear permits a detailed characterization of the sound-elicited ionic currents because whole-cell patch-clamp recordings can be conducted during concurrent stimulation by airborne sound in an intact ear (Warren and Matheson, 2018); an approach not possible in other hearing models. In response to acoustic overstimulation we measured physiological changes in the sound-evoked displacements of the tympanum, electrical responses from the auditory nerve, ionic currents in individual auditory neurons, and relative abundances of the putative sound-activated ion channel genes, *nompC*, *nanchung* and *inactive*.

## Materials and Methods

### 

#### Locust husbandry

Desert locusts (*Schistocerca gregaria*) were reared in crowded conditions (phase gregaria) on a 12 h light/dark cycle at 36.25°C. Locusts were fed on a combination of fresh wheat and bran *ab libitum*. Male locusts between 10 and 20 d post-imaginal molt were used for all experiments. We used new locusts for each set of experiments. We are willing and open to share our locust strains with other research groups.

#### Noise exposure and acoustic stimulation

The wings of all locusts were cut off at their base to increase noise exposure of the conditioning tone to their tympanal ears, which are otherwise covered by their wings. Up to 20 locusts, for both the noise-exposed group and the control group, were placed in a cylindrical wire mesh cage (8 cm diameter, 11 cm height). Both cages were placed directly under a speaker (Visaton FR 10 HM 4 OHM, RS Components). For the noise-exposed group only, the speaker was driven by a function generator (Thurlby Thandar Instruments TG550, RS Components) and a sound amplifier (Monacor PA-702, Insight Direct) to produce a 3 kHz tone at 126 dB sound pressure level (SPL), measured at the top of the cage where locusts tended to accumulate. This tone was played continuously for 24 h for the noise-exposed group. The control group was housed in an identical cage with a silent speaker for 24 h. All mechanical and electrophysiological recordings were performed within 8 h of cessation of noise-exposure. SPLs were measured with a microphone (Pre-03 Audiomatica, DBS Audio) and amplifier (Clio Pre 01 preamp, DBS Audio). The microphone was calibrated with a B&K Sound Level Calibrator (CAL73, Mouser Electronics). The locust ear was stimulated with the same speaker and amplifier as described for hook electrode recordings at 3 kHz with a rise and fall time of 2 ms. For intracellular recordings from individual auditory neurons the speaker was driven by a custom made amplifier controlled by a EPC10-USB patch-clamp amplifier (HEKA Elektronik) controlled by the program Patchmaster (v2 × 90.2, HEKA Elektronik) running under Microsoft Windows v7.

#### Biomechanical measurements of the tympanum with laser Doppler vibrometry

For *in vivo* measurements the locusts wings and hind legs were cut off and locusts were fixed so that their tympanum was perpendicular to the micro-scanning Laser Doppler Vibrometer (PSV 300, Polytec) with a close up unit (OFV 056). A loudspeaker (ESS Air Motion Transformer) was placed at least 10 cm away, to avoid operating in the near field. A microphone (Bruel and Kjaer 4138) was positioned to measure the sound pressure at the tympanal membrane.

For *ex vivo* measurements whole ears, including Müller's organ attached to the internal side of the tympanum, were dissected from the first abdominal segment, by cutting around the small rim of cuticle surrounding the tympanum with a fine razor blade. Trachea and the auditory nerve (Nerve 6) were cut with fine scissors (5200-00, Fine Science Tools), and the trachea and connective tissue removed with fine forceps. The ear was secured, inner side up, into a 2-mm-diameter hole in a Perspex divider (to download the locust ear holder file for 3D printing: www2.le.ac.uk/departments/npb/people/bw120) using an insect pin pushed through the anterior rim of cuticle and into 2 mm of Sylgard (184 Silicone Elastomer, Dow Corning) on the base of a 30-mm-diameter petri dish. The tympanum in its holder was positioned at an angle of 30° off vertical to observe group-III neurons of Müller's organ from above. A watertight seal was made between the ear cuticle and the divider hole with dental glue (Protemp 4, 3 M ESPE) and nerve 6 was secured into the glue at the ventral border of the tympanum.

#### *In vivo* hook electrode recordings from auditory nerve six

Locusts were secured ventral side up in plasticine. A section of the second and third ventral thoracic segment was cut with a fine razor blade and removed with fine forceps. Tracheal air sacks were removed to expose nerve six and the metathoracic ganglia. Hook electrodes constructed from silver wire 18 μm diameter (AG549311, Advent Research Materials) were hooked under the nerve and the nerve was lifted out of the hemolymph. Signals were amplified 10,000 times by a differential amplifier (Neurolog System) then filtered with a 500 Hz high-pass filter and a 50 kHz low-pass filter. This amplified and filtered data were sampled at 25 kHz by Spike2 v8 software running on Windows v10. To count the number of nerve potentials for hook electrode recordings we counted each potential >50 μV. We did this for all locusts irrespective of the treatment of the locust for an objective measure of hearing function and all locusts were used for analysis. N.B. the locust treatment was blinded to the experimenter until all data were collected and analyzed. For measurements of tone-evoked spike latency we only measured latencies where there was a clear tone-elicited nerve potential >50 μV. Thresholds were determined as the minimum SPL necessary to get an increase in the nerve response. An increase in the nerve response, or threshold, was classified when the nerve potential count is sequentially higher for each of the next three highest SPL tones (i.e., the threshold was 40 dB SPL when the number of nerve potentials was higher at 50 dB SPL, then higher again for 60 dB SPL, and higher still for 70 dB SPL).

#### Dissection of Müller's organ and isolation of group-III auditory neurons and discovery of a muscle through which the nerve of Müller's organ loops

We focused our analyses on group-III auditory neurons because they form the majority of auditory neurons of the Müller's organ (∼46 of ∼80; Jacobs et al., 1999), they are the most sensitive auditory neurons of the Müller's organ (Römer, 1976) and whole-cell patch-clamp recordings were only possible from these neurons (Warren and Matheson, 2018). For intracellular patch-clamp recordings from individual auditory neurons the abdominal ear was excised and placed into a preparation dish as explained (see *Biomechanical measurements of the tympanum with laser Doppler vibrometry*). This preparation allowed perfusion of saline to the internal side of the tympanum, necessary for water-immersion optics for visualizing Müller's organ and the auditory neurons to be patch-clamped, and concurrent acoustic stimulation to the dry external side of the tympanum. The inside of the tympanum including Müller's organ was constantly perfused in extracellular saline.

To expose group-III auditory neurons for patch-clamp recordings, a solution of collagenase (0.5 mg/ml) and hyaluronidase (0.5 mg/ml; C5138, H2126, Sigma-Aldrich) in extracellular saline was applied onto the medial-dorsal border of Müller's organ through a wide (12 μm) patch pipette to digest the capsule enclosing Müller's organ and the Schwann cells surrounding the auditory neurons. Gentle suction was used through the same pipette to remove the softened material and expose the membrane of group-III auditory neurons. The somata were visualized with an upright microscope (BH-2, Olympus) using a water-immersion objective (W Plan- APOCHROMAT, 40×, 1.0 numerical aperture, 2.5 mm working distance; Zeiss) and differential interference contrast optics.

The auditory nerve of the Müller's organ passes through a striated muscle that is anchored on the lateral side of the tympanic rim, near a spiracle opening. We hypothesize that this muscle could contract, pulling Müller's organ nerve and tympanum taught, in response to loud noise to prevent excessive sound induced vibrations.

#### Electrophysiological recordings and isolation of the transduction current

To quantitatively record tone-evoked currents and spikes from individual auditory neurons, the ear was excised from the locust and Müller's organ was immersed in saline. This is necessary to gain access to, image and record from individual auditory neurons through whole-cell patch-clamp intracellular recordings (Warren and Matheson, 2018). Electrodes with tip resistances between 3 and 4 MΩ were fashioned from borosilicate class (0.86 mm inner diameter, 1.5 mm outer diameter; GB150-8P, Science Products) with a vertical pipette puller (PP-830, Narishige). Recording pipettes were filled with intracellular saline containing the following (in mm): 190 K-aspartate, 4 NaCl, 2 MgCl_2_, 1 CaCl_2_, 10 HEPES, 10 EGTA. To block K^+^ channels necessary for isolation the transduction current 20 mm tetraethylammonium chloride (TEA) was added to the intracellular saline, K-aspartate was reduced to 170 mm to maintain the same osmolality. To isolate the transduction current we also blocked spikes with 90 nm tetrodotoxin (TTX) in the extracellular saline. During experiments, Müller's organs were perfused constantly with extracellular saline containing the following (in mm): 185 NaCl, 10 KCl, 2 MgCl_2_, 2 CaCl_2_, 10 HEPES, 10 trehalose, 10 glucose. The saline was adjusted to pH 7.2 using NaOH. The osmolality of the intracellular and extracellular salines were 417 and 432 mOsm, respectively.

Dihydrostreptomycin sesquisulfate, 50 μm (D7253, Sigma-Aldrich) was used to block mechanotransduction ion channels. Dihydrostreptomycin sesquisulfate was perfused at least 15 min before recordings. Whole-cell voltage-clamp recordings were performed with an EPC10-USB patch-clamp amplifier (HEKA Elektronik) controlled by the program Patchmaster (v2 × 90.2, HEKA Elektronik) running under Microsoft Windows v7. Electrophysiological data were sampled at 50 kHz. Voltage-clamp recordings were low-pass filtered at 2.9 kHz with a 4-pole Bessel filter. Compensation of the offset potential was performed using the “automatic mode” of the EPC10 amplifier and the capacitive current was compensated manually. The calculated liquid junction potential between the intracellular and extracellular solutions was also compensated (15.6 mV for normal saline and 13.5 mV for TTX and TEA saline; calculated with Patcher's-PowerTools plug-in; www3.mpibpc.mpg.de/groups/neher/index.php?page=software). Series resistance was compensated between 50 and 70% with a time constant of 100 μs.

#### Staining and confocal microscopy

To stain group-III auditory neurons, recording electrodes were filled with neurobiotin (1% m/v, SP-1120, Vector Laboratories) dissolved in intracellular saline. To aid diffusion of neurobiotin into the neurons a positive current of ∼200 pA was injected for ∼30 min. Directly after staining, Müller's organs were fixed overnight at 5°C in 4% paraformaldehyde (P6148, Sigma-Aldrich) dissolved in PBS. Müller's organs were then washed three times in PBS then gently shaken at room temperature for 20 min in a mixture of collagenase (0.5 mg/ml) and hyaluronidase (0.5 mg/ml; C5138 and H2126, Sigma-Aldrich). They were washed three times in PBS (3 × 10 min) then gently shaken at room temperature in 0.2% m/v Triton X-100 dissolved in PBS (2 × 60 min). Müller's organs were then gently shaken in 20 μg/ml Dylight 488 streptavidin (SA-5488, Vector Laboratories) and 0.05 mg/ml DAPI (D9542, Sigma-Aldrich) in PBS overnight at 5°C. During this time the fluorescent streptavidin binds very tightly to the fixed neurobiotin to specifically stain the recorded neurons. After this overnight incubation the Müller's organs were washed three times in PBS (3 × 10 min), dehydrated in an ethanol series and cleared in methyl salicylate (M6752, Sigma-Aldrich).

Fluorescence images (pixel size 0.31 μm^2^, *z* stacks of 0.31 μm) were captured with a confocal microscope (FV1000 CLSM, Olympus) equipped with Plan-UPlanSApo 10× (0.4 numerical aperture) and 20× (0.75 numerical aperture) lenses. Fluorescence emission of Dylight 488 was collected through a 505–530 nm bandpass filter. Confocal images were adjusted for contrast and brightness, overlaid and stacked in ImageJ (v1.51, National Institutes of Health). The ImageJ plugin Simpler Neurite Tracer was used to determine the distance from the center of the soma to the dendrite dilation (see Fig. 3*C*).

#### RNA extraction, sequencing, and transcriptome analysis

A total of 320 Müller's organs from 160 control locusts (2 ears per locust) and 320 Müller's organs from 160 noise-exposed locusts were extracted by grasping the Müller's organ through the tympanum with fine forceps and pulling it out. Müller's organ RNA extraction took place within 4 h after the 24 h noise-exposure was finished. Müller's organs were placed in an Eppendorf tube submerged in liquid nitrogen. RNA was extracted and then treated with DNase using a RNAqueous kit (AM1931, ThermoFisher). RNA was shipped in dry ice to Beijing Genomics Institute (Hong Kong) for sequencing. The samples were quality checked and had ribonucleic acid (RNA) integrity values of 8.7 and 8.4 for control and noise-exposed samples, respectively. The two samples were sequenced using Illumina HiSeq 2000 using a 100 paired-ends module. We obtained 186.1 million clean reads for RNA extracted from the Müller's organs of noise-exposed locusts and 185.9 million clean reads from the Müller's organs of control locusts. We analyzed the RNA reads and quantified expression levels (abundances) for the three genes that compose the two mechanotransduction ion channel candidates in insects: *nompC*, *nanchung*, and *inactive* (*nanchung* and *inactive* together code a single heteromeric Nanchung-Inactive ion channel) and the two housekeeping genes *actin* and *GAPDH*. Initial cDNA reads were groomed to have 80 or more contiguous nucleotides with Phred score >19 to give a final database of ∼100 million pairs of reads. Sequences-of-interest were identified by searching all possible translations of reads from the control transcriptome versus amino acid sequences of published insect genes, including *Drosophila melanogaster*, using BLOSUM matching matrices (Henikoff and Henikoff, 1993). Identified reads-of-interest were extended by the transcriptome walking algorithm (French, 2012) using an initial minimum overlap of 60 nt. Walking was always continued to identify the complete protein coding sequence, including both START and STOP codons.

Relative abundances of transcribed mRNA sequences in the two tissues were estimated by searching each complete groomed transcriptome library for reads matching the reading frame of each gene, using the criterion of at least 90/100 identical nucleotide matches to score each read as derived from the gene. Matching reads as a fraction of total reads counted were then normalized by gene length and expressed as abundance relative to the most abundant *actin* gene in the control tissue.

#### Experimental design and statistical analysis

For all recordings we used male *Schistocerca gregaria* from the Leicester “Locust Labs” laboratory stock. Throughout the manuscript *n* refers to the number of recorded neurons and *N* refers to the number of Müller's organ preparations used to achieve these recordings (i.e., *n* = 10, *N* = 6 means that 10 neurons were recorded from 6 Müller's organs). All *n* numbers are displayed on the figures for clarity. The reason for variation in *n* numbers for some figures (see Fig. 4*D*) is because noise-exposed auditory neurons failed to produce a response at lower SPLs compared with auditory neurons from control locusts. The spread of the data are indicated by 1 SD because the SD indicates the spread of the data, unlike SE. Median and Q1 and Q3 are displayed by bars when individual measurements are plotted. The treatment of the locust: noise-exposed or control, remained blinded to the experimenter until data analysis was completed. To test for differences and interactions between control, noise-exposed, and streptomycin-treated locusts we used either a linear model (LM) or linear mixed-effects model (LMEM), with decibel SPL and treatment as fixed effects, and Locust ID as a random intercept, when repeated measures are reported. We used a Wilcoxon test when comparing thresholds of hearing for hook electrode recordings (see Fig. 2*E*, inset), because these data were not continuous. Models were fitted in R (v3.4.3) with the package LME4 (Bates et al., 2015). The test statistic for these analyses (*t*) are reported with the degrees of freedom (subscript) and *p* value, which are approximated using Satterthwaite equation (lmerTest package; Kuznetsova et al., 2017). We report Cohen's *d* effect size for significant differences. Curves where fitted to the data using MATLAB (vR2018a, MathWorks). The shape of the data was described by fitting Boltzmann voltage equation using nonlinear least-squares method. For each curve fit the goodness of fit is indicated by *R*^2^. For testing differences between the latency of tone-evoked spikes (see Figs. 4*D*, 6*D*) we analyzed the highest 5 SPLs because: (1) the higher *n* numbers at these louder SPLs provided more rigorous power, and (2) the latencies at these higher SPLs reached a plateau and therefore did not markedly change.

To measure the latency to transduction current onset we plotted a linear fit to the current trace 150 ms after tone onset of the adapted transduction current. A second line was plotted between the peak transduction current and tone onset. Latency was taken as the intercept between these two lines. The discrete depolarizations added significantly to the noise, hence why an average of the current was taken.

This is the first study to measure the effects of noise exposure on the locust's auditory system. Thus, we had no a priori effect size for power calculations but we will able to use the effect sizes reported here for power calculations for future studies using the locust ear as a model for hearing loss.

## Results

### Biomechanics of the tympanum

**Figure 1.**
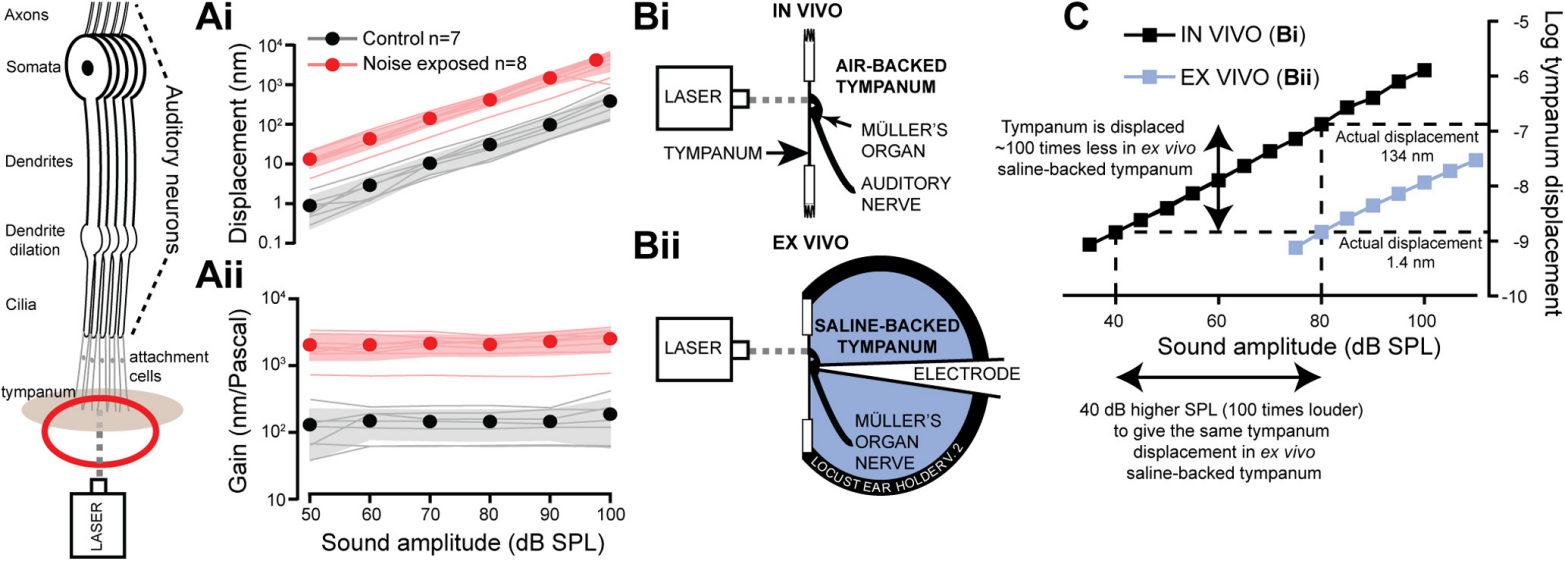
*In vivo* Doppler laser measurements of tympanal displacements shows increased displacements and gain for noise-exposed locusts. ***Ai***, The displacement of the tympanum was higher for noise-exposed locusts. ***Aii***, The gain, nanometer displacement per Pascal, was also higher for noise-exposed locusts. ***Bi***, The experimental setup for *in vivo* recording from an intact ear. ***Bii***, Experimental setup for *ex vivo* recording with a saline-backed tympanum, necessary for intracellular recordings from individual auditory neurons. ***C***, Comparison of tympanal displacements in an *in vivo* and *ex vivo* preparation backed by air and saline, respectively. A 40 dB louder tone is required to move the tympanum by the same amount when backed by saline (*ex vivo*) compared with air (*in vivo*).

We first focused our analyses on *in vivo* measures of hearing impairment after auditory overexposure. Noise-exposed locusts were exposed to a 126 dB SPL 3 kHz tone for 24 h, whereas control locusts were held in identical conditions but under a silent speaker. For an *in vivo* measure of hearing impairment, we exploited the accessible nature of the locust ear to measure *in vivo* tone-evoked vibrations from the external surface of the tympanum, directly opposite where group III auditory neurons are attached on the inside of the tympanum. Group III auditory neurons form the majority ∼46 of ∼80 neurons in Müller's organ and are broadly tuned to 3 kHz (Jacobs et al., 1999; Warren and Matheson, 2018). For multiple auditory systems across phyla (including humans) the health of the auditory receptor cells can be surveyed from the sounds produced by mechanical vibrations of the tympanum (Kemp, 2002). Therefore, we measured the displacement and mechanical gain of the tympanum. Mechanical gain is the amplitude of vibrations as a function of sound amplitude. The displacement of the tympanum and its mechanical gain was increased across SPLs from 50 to 100 dB SPL for noise-exposed locusts (Fig. 1*Ai*, Displacement of tympanum: Linear Mixed Effects Model (LMEM) *t*_(86.0)_ = 4.829, *p* < 0.0001, effect size: *d* = 0.75896; Fig. 1*Aii*, Mechanical gain, LMEM: *t*_(79.18)_ = −3.432, *p* = 0.0009, effect size: *d* = 3.44 854; Control: *n* = 7, *N* = 7; Noise-exposed: *n* = 8, *N* = 8).

To quantitatively record tone-evoked currents and spikes from individual auditory neurons the ear was excised from the locust and Müller's organ was immersed in saline. This is necessary to gain access to, image and record from individual auditory neurons through whole-cell patch-clamp intracellular recordings (Warren and Matheson, 2018). We quantified the reduction in tone-evoked tympanal vibrations when the tympanum is backed by water; as opposed to its tracheal air backing when *in vivo* (Fig. 1*Bi*,*Bii*). We found that vibrations of the tympanum were 100× lower when the tympanum was backed by saline or, put another way; we required a tone 40 dB louder to move the tympanum by the same amount when backed by saline as opposed to air (Fig. 1*C*).

### *In vivo* electrophysiology

**Figure 2.**
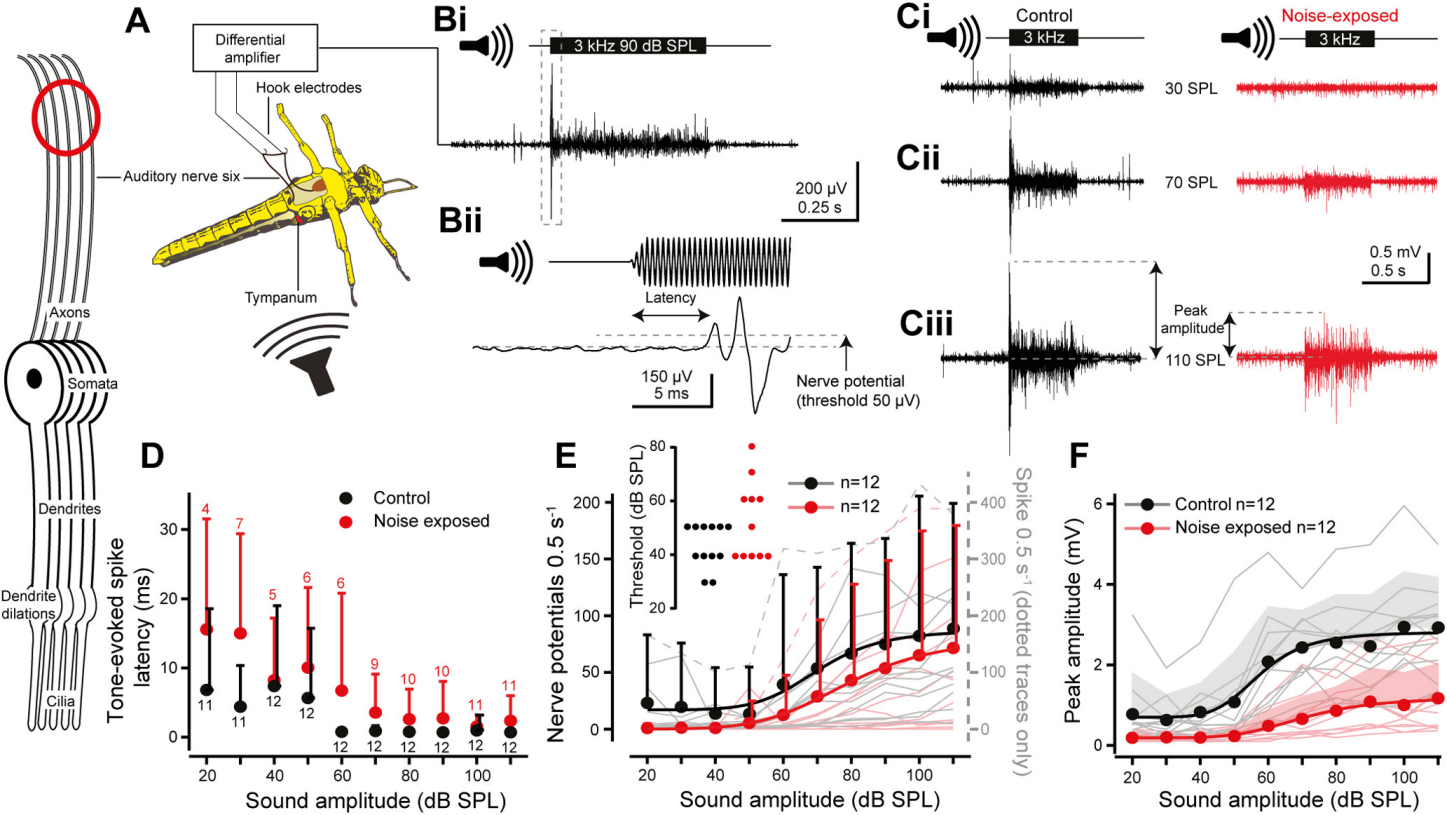
*In vivo* hook electrode recordings from the auditory nerve (six). Neuron schematic on left (red circle) indicates where the electrical signals were recorded; from the auditory nerve. ***A***, Schematic showing the vivisection and placements of the hook electrodes under the auditory nerve. ***Bi***, Example recording using a 3 kHz tone at 90 dB SPL with (***Bii***) an expanded view (dotted box from ***Bi***) to show latency to first nerve potential and the 50 µV threshold used to count nerve potentials. ***C***, Nerve responses for a 0.5 s 3 kHz tone at (***Ci***) 30, (***Cii***) 70, and (***Ciii***) 110 dB SPL (peak amplitude calculation for ***F*** is shown with double-headed arrows). ***D***, Quantification of the latency to first nerve potential in response to a 3 kHz tone for control and noise-exposed locusts. Means are plotted as circles, positive SD is plotted as error bars. One auditory nerve from a noise-exposed locust had an extremely high spontaneous spike rate and was not included in the latency analysis. ***E***, Quantification of the number of tone-evoked nerve potentials >50 µV, which increased for louder sound amplitudes. Nerve potential counts are well fitted with Boltzmann equations (solid lines). Means are plotted as circles, positive SD is plotted as error bars (the error bars for noise-exposed means are offset, right, for figure clarity). Nerve potential counts from individual locusts are plotted as thin shaded lines. One auditory nerve from a noise-exposed locust did not show any response to even the highest SPLs. N.B. Two individual particularly high neve potential counts, included in the analysis, are plotted as dotted lines on the right axis. Inset, The thresholds SPLs for all recordings. ***F***, The peak amplitude response of control locusts was higher than noise-exposed locusts at higher SPLs. The peak response increased for higher sound amplitudes and was well fitted with a Boltzmann equation (solid lines).

Next, we investigated the effect of noise-exposure on the ability of auditory neurons to produce sound-evoked spikes. To accomplish this, we recorded tone-evoked nerve potentials from the auditory nerve (nerve six) with hook electrodes in an *in vivo* preparation that left the first abdominal segment, with its bilateral ears, intact (Fig. 2*A*). The nerve potentials (Fig. 2*Bi*) are a read-out of the population activity of Müller's organ auditory neurons. The majority of the auditory neurons are broadly tuned to 3 kHz (Jacobs et al., 1999), so we used a 3 kHz tone to elicit nerve potentials, which were recorded as multiphasic extracellular potentials with a short delay (Fig. 2*Bi*,*Bii*). An increased sound amplitude elicited an increase in the nerve potentials (Fig. 2*Ci*–*Ciii*).

Noise-exposed locusts replicated other hearing models with delayed spike generation in their auditory nerves compared with control locust ears (Fig. 2*D*; LMEM: *t*_(213.9)_ = 0.457 *p* = 0.001, effect size *d* = 0.666374). Both control and noise-exposed locusts' spike latency were reduced at higher sound amplitudes. Tone-evoked nerve potentials, >50 µV in amplitude, were positively correlated with sound amplitude, which was well fitted with a Boltzmann equation (Fig. 2*E*, solid lines; Control *R*^2^ = 0.962; Noise-exposed *R*^2^ = 0.997) but noise-exposure had no significant decrease on the number of nerve potentials (LMEM *t*_(27.27)_ = 0.690, *p* = 0.496). We determined threshold responses to 3 kHz tones (Fig. 2*E*, inset), which was not different between control and noise-exposed locusts (Wilcoxon test, *W* = 43, *p* = 0.147). We also measured the peak amplitude of the nerve potentials (Fig. 2*Ciii*, double-headed arrows), which is the summated response of multiple spikes, typically elicited at tone onset. The peak amplitude was well fitted with a Boltzmann relationship (Control *R*^2^ = 0.973; Noise-exposed *R*^2^ = 0.986), and the peak amplitude of noise-exposed locusts was below that of their control counterparts, but only for higher SPLs (Fig. 2*F*; LMEM: Treatment x SPL interaction, *t*_(649.0)_ = 10.00 *p* < 0.0001, effect size: *d* = 1.138042).

### Auditory neuron morphology, electrical properties, and transduction current

**Figure 3.**
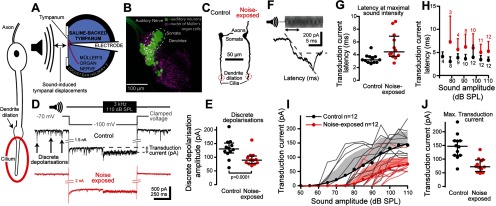
Intracellular whole-cell patch-clamp recordings of auditory neurons, their spontaneous and tone-evoked transduction ion channel activity and neuron morphology. All spontaneous and tone-evoked transduction currents occur in the auditory neurons' cilium highlighted by a red circle on the neuron schematic on the left. ***A***, Schematic showing experimental setup for intracellular patch-clamp recordings from Müller's organ housed on the internal surface of the tympanum perfused with saline. The outside of the tympanum is acoustically driven by airborne sound by a speaker. ***B***, Double staining of the nuclei of cells of Müller's organ (magenta; DAPI) and all auditory neurons with whole nerve backfill using neurobiotin/Dylight 488 streptavidin (green). Group-III auditory neurons are highlighted by a white dotted circle. ***C***, A neurobiotin-streptavidin staining of two group-III auditory neurons *in situ* reveals the dendrite dilation and apical cilium. ***D***, Sound stimulation, voltage-clamp and recording protocol used to maximize the transduction current during intracellular patch-clamp recordings. A 3 kHz tone at 110 dB SPL (top trace) was used to stimulate group-III auditory neurons at their most sensitive frequency. The neurons were voltage-clamped to −100 mV (gray trace) to increase the electrochemical driving force. The extracellular saline contained 90 nm TTX and intracellular pipette solution contained 20 mm TEA to block sodium and potassium conductances and the spikes they facilitate. Discrete depolarizations (arrows) and the tone-evoked transduction currents are reduced in noise-exposed auditory neurons (red) compared with control (black). ***E***, The amplitude of discrete depolarizations is reduced for auditory neurons from noise-exposed ears. The maximum six discrete depolarizations were averaged for each locust during the −100 mV voltage clamp. ***F***, Example showing calculation of the latency of the tone-evoked transduction current. ***G***, Latency of the transduction current was delayed for noise-exposed auditory neurons at 110 dB SPL and (***H***) across sound intensities. ***I***, The transduction current increased with louder sound amplitudes, which was well fitted with a Boltzmann equation (solid lines). Mean is plotted as circles, positive SD is plotted as shaded area, transduction current amplitude from individual auditory neurons are plotted as thin shaded lines (Control: *n* = 12, *N* = 6; Noise-exposed: *n* = 12, *N* = 7). ***J***, The maximal transduction current was significantly reduced at the maximal sound intensity of 110 dB SPL.

We measured auditory neuron morphology, characterized their electrical properties and measured their sound-elicited currents through whole-cell patch-clamp recordings from individual neurons from excised ears. Group III neurons in Müller's organ (Fig. 3*A*–*C*) have a long ∼100 µm dendrite whose length and diameter was not different between control and noise-exposed locusts (Fig. 3*C*; Table 1). In addition, there were no large differences in the membrane potential, membrane resistance and capacitance between noise-exposed and control auditory neurons (Table 1).

**Table 1.** Morphologic and electrophysiological properties of auditory neurons

	Dendrite length, µm	Dendrite diameter at midpoint, µm	Resting potential, mV	Membrane resistance, MΩ	Capacitance, pF
Control	114 ± 13 (*n* = 8)	3.2 ± 0.74 (*n* = 8)	60 ± 8 (*n* = 11)	50 ± 47 (*n* = 12)	25 ± 6 (*n* = 12)
Noise-exposed	120 ± 15 (*n* = 8)	3.1 ± 0.60 (*n* = 8)	57 ± 5 (*n* = 9)	54 ± 45 (*n* = 12)	22 ± 5 (*n* = 12)

There were no significant differences between the morphologic or electrophysiological properties of noise-exposed and control auditory neurons (LM: *p* = 0.464, 0.651, 0.362, 0.7826, 0.164; *t* = 24.51, 0.462, −0.935, −0.280, 1.441, respectively). These measurements were recorded from spiking neurons with no TTX and TEA in the extracellular or intracellular saline. Only neurons with a resting membrane potential at least −50 mV were used to compare resting potentials.

We isolated and optimized the transduction current at the distal ciliated end of the auditory neuron using pharmacology, voltage protocols and the optimal sound stimulus (Fig. 3). We recorded smaller discrete depolarizations in the auditory neurons of noise-exposed locust auditory neurons compared with control (Fig. 3*D*,*E*; LM *t*_(142)_ = 6.524, *p* < 0.0001, effect size: *d* = 1.087299). Discrete depolarizations are assumed to be transient stochastic opening of mechanotransduction ion channels shown both in insects (Hill, 1983b; Warren and Matheson, 2018) and vertebrate auditory receptors (Pan et al., 2013; Beurg et al., 2015). There was a significant delay in the generation of the transduction current (Fig. 3*F*) in noise-exposed locusts compared with their control counterparts at the maximal sound amplitude of 110 dB SPL (Fig. 3*G*) and across sound amplitudes (Fig. 3*H*; LMEM *t*_(109.38)_ = 2.225, *p* = 0.0281, effect size: *d* = 0.54 342). The transduction current increased with increased sound amplitude. This dependence was well fitted with a Boltzmann equation (Control: *R*^2^ = 0.990, Noise-exposed: *R*^2^ = 0.996) with a clear reduction in the amplitude of the transduction current for noise-exposed locust auditory neurons (Fig. 3*I*; LMEM *t*_(138.93)_ = −13.92, *p* < 0.0001, effect size: *d* = 0.69 729). The largest difference was at their maximal transduction currents, which were 146 ± 45 and 78 ± 25 pA for control and noise-exposed locusts, respectively (Fig. 3*J*).

### Tone-evoked dendritic spikes

**Figure 4.**
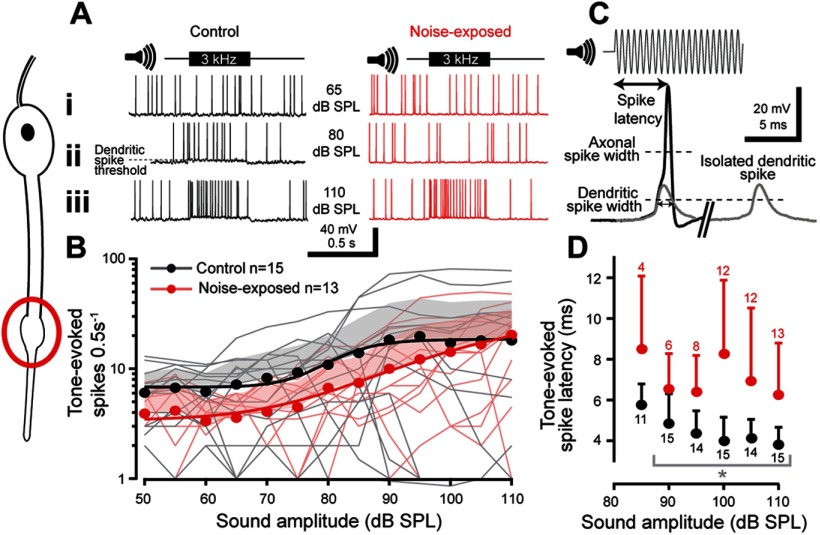
Tone-evoked spikes were recorded in current-clamp mode with whole-cell patch-clamp recordings from auditory neurons from control and noise-exposed locust ears. Dendritic spikes are elicited in the distal part of the dendrite, with its presumed site highlighted by the red circle (left). ***Ai*–*Aiii***, Example recordings from control and noise-exposed auditory neurons when played a 0.5 s 3 kHz tone at 65, 85, and 110 dB SPL. ***Aii***, The gray dotted line indicates the potential at which a dendritic spike threshold was calculated. ***B***, The number of tone-evoked spikes was higher for louder sound amplitudes, which was well fitted by a Boltzmann equation (solid lines). Mean is plotted as circles, positive SD is plotted as shaded area, spike counts from individual locusts are plotted as thin shaded lines (Control: *n* = 15, *N* = 8; noise-exposed: *n* = 13, *N* = 12). ***C***, Example recording showing how the latency of tone-evoked spike was measured and a rare isolated dendritic spike (gray) later in the same recording, which is overlaid on the larger axonal spike. Dotted lines indicate where the axonal spike half-width and dendritic spike half-width were measured. ***D***, The latency to first spike was slower for auditory neurons from noise-exposed locusts (red) compared with controls (black) across sound amplitudes. Means are plotted as circles; positive SD is plotted as error bars. Gray brackets with asterisk donate the recordings that were statistically tested (for justification, see Materials and Methods, Experimental design and statistical analysis).

The transduction potential depolarizes the cilium and adjacent distal dendrite of the auditory neuron, and if large enough, triggers a small dendritic spike that travels to the soma to trigger a larger axonal spike in the axon hillock (Warren and Matheson, 2018). Because of its morphology the assumed dendritic spike initiation site is the dendrite dilation that sits ∼5 µm below the cilium (Fig. 3*C*). To measure changes in tone-evoked dendritic spikes we used whole-cell patch-clamp recordings in current-clamp mode. In the absence of sound stimulation spontaneous spikes were recorded that tended to be lower in noise-exposed auditory neurons (Control: 5.8 ± 0.9 spikes per 0.5 s, *n* = 15; Noise-exposed: 3.4 ± 0.7 spikes per 0.5 s, *n* = 13; LM: *t*_(25.0)_ = 7.047, *p* < 0.0001, effect size: *d* = 0.667). The number of tone-evoked spikes increased for higher SPLs (Fig. 4*A*; LMEM *t*_(333.0)_ = 7.070, *p* < 0.0001). In response to a >60 dB SPL 3 kHz tone, tone-evoked spikes were triggered that closely followed a Boltzmann relationship with sound amplitude (Fig. 4*B*; Control: *R*^2^ = 0.965; Noise-exposed: *R*^2^ = 0.992). The number of tone-evoked spikes tended to be (but was not significantly) less for noise-exposed auditory neurons (Fig. 4*B*; LMEM *t*_(169.38)_ = 0.975, *p* = 0.3309). The latency to first spike for all neuron types decreased for increased SPLs (Fig. 4*C*,*D*). The time to first spike was delayed for noise-exposed auditory neurons when compared with control auditory neurons across SPLs (LM *t*_(122)_ = 7.154, *p* < 0.0001, effect size: *d* = 1.30 566). We measured the width of spikes at two heights (Fig. 4*C*) to measure possible changes in both the smaller dendritic spikes and the larger axonal spikes that they trigger. To measure axonal spike width we measured at half-height (Fig. 4*C*; axonal spike half-width). To infer a measure of dendritic spike width we measured at half of the height of the dendritic spike (Fig. 4*C*; dendritic spike half-width) The height of the dendritic spike was determined by taking the start of the maximum acceleration of the spike's depolarization as the dendritic spike height. The axonal and dendritic spike width was not different between noise-exposed and control locusts (Control axonal spike width: 0.81 ± 0.24, *n* = 15; Noise-exposed axonal spike width: 0.74 ± 0.16, *n* = 12; LM: *t*_(25.0)_ = 0.53 *p* = 0.601; Control dendritic spike width: 1.48 ± 0.44, *n* = 9; Noise-exposed dendritic spike width: 1.37 ± 0.23, *n* = 8; LM: *t*_(15.0)_ = 0.641, *p* = 0.531). For each auditory neuron we measured the tone-elicited potential that produced an increase in the number of spikes above the background spontaneous spike rate; the dendritic spike threshold (Fig. 4*Aii*, dotted line). The dendritic spike threshold was similar for control and noise-exposed locust auditory neurons (Control dendritic spike threshold: 67.1 ± 5.1 mV, *n* = 14; Noise-exposed dendritic spike threshold: 68.9 ± 5.9 mV, *n* = 12; LM: *t*_(24.0)_ = 0.822, *p* = 0.419).

### Current-injected axonal spikes

**Figure 5.**
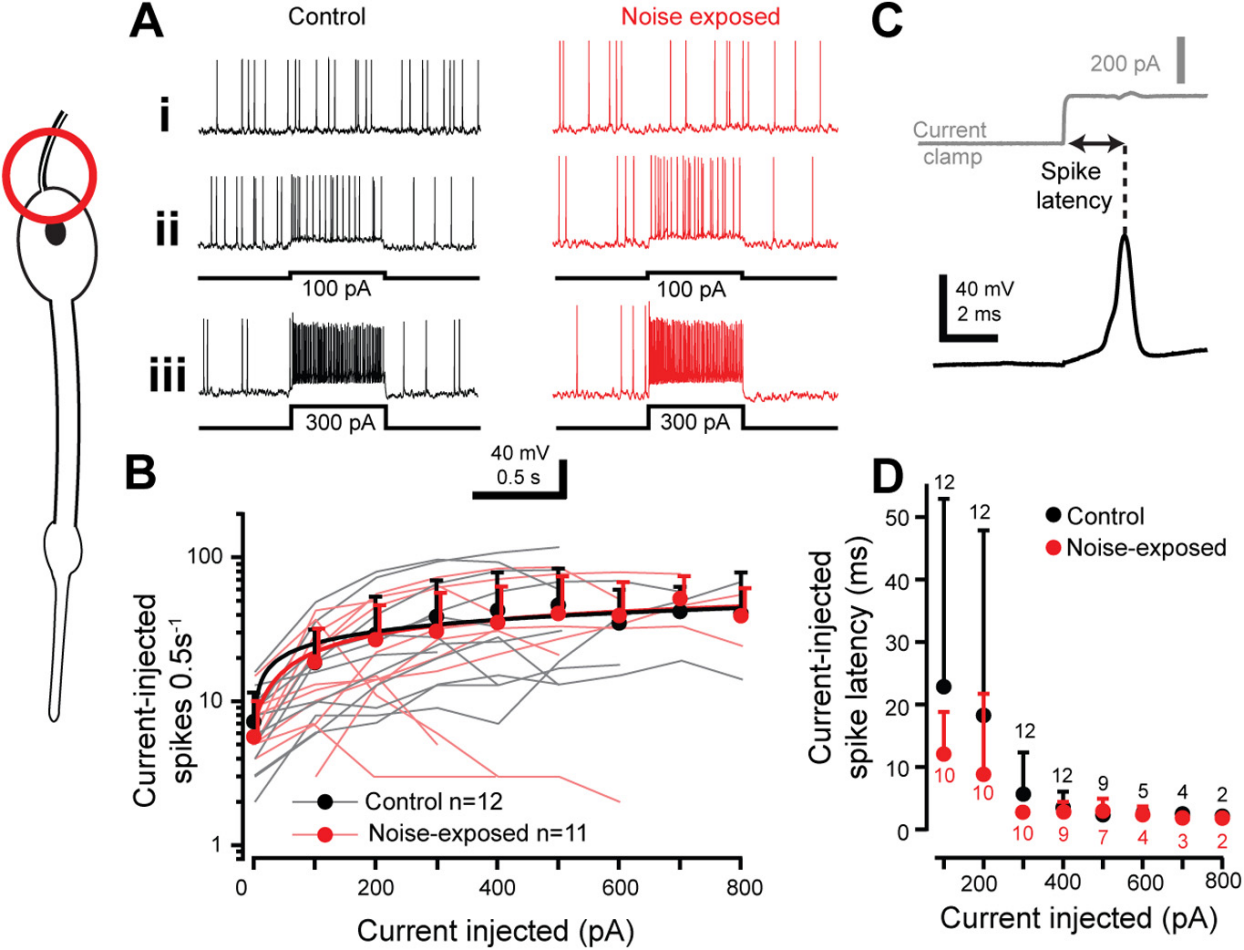
Axonal spikes were elicited through current injection into the auditory neuron somata of control and noise-exposed locust ears. Axonal spikes are assumed to be triggered in the axon hillock, red circle (left). ***A***, Example recordings showing (***Ai***) spontaneous spikes and (***Aii***) spikes in response to 100 pA current injection and (***Aiii***) spikes in response to 300 pA current injection. ***B***, The number of spikes triggered by current injection was fitted by a power law (solid lines) and was not different for auditory neurons from noise exposed (red) or control locusts (black) across all current injections. Means are plotted as circles; positive SD is plotted as error bars (the error bars for noise-exposed means are offset, right, for figure clarity). Current-elicited spike number from individual auditory neurons are plotted as thin shaded lines (Control: *n* = 12, *N* = 9; Noise-exposed: *n* = 11, *N* = 10). ***C***, An example measurement of the current-injected spike latency. ***D***, Current-injected spike latency across and range of injected currents, which were not different between control and noise-exposed locusts. Recordings were lost at high current injections, hence the lower *n* numbers at high current injections. Means are plotted as circles; positive SD is plotted as error bars (1 neuron from the noise-exposed group was not analyzed for latency due to its high spontaneous spike rate).

Auditory neurons of Orthopteran's have two spike types: a dendritic spike that propagates to the soma and axonal spikes that are elicited by dendritic spikes and carry auditory information to the central nervous system. Next, we focused our analyses on axonal spikes to test if changes in axonal spikes contribute to auditory deficits after noise exposure. To elicit axonal spikes (with their presumed spike initiation site in the axon hillock) (Fig. 5*A*, red circle) current was injected into the soma through the patch electrode (Fig. 5*A*). Current injected into either control or noise-exposed auditory neurons resulted in the generation of axonal spikes (Fig. 5*Ai*–*Aiii*). The spike rate varied as a power law of the injected current, with no difference between control and noise-exposed auditory neurons (Fig. 5*B*; LMEM: *t*_(28.38)_ = −0.054, *p* = 0.9575). The spike latency (Fig. 5*C*) decreased for increasing current injections (Fig. 5*D*) and there was no meaningful difference in latency between control and noise-exposed auditory neurons (Fig. 5*D*; LMEM: *t*_(58.24)_ = −1.273, *p* = 0.208).

### Streptomycin block of the mechanotransduction channels mimics noise-exposure

**Figure 6.**
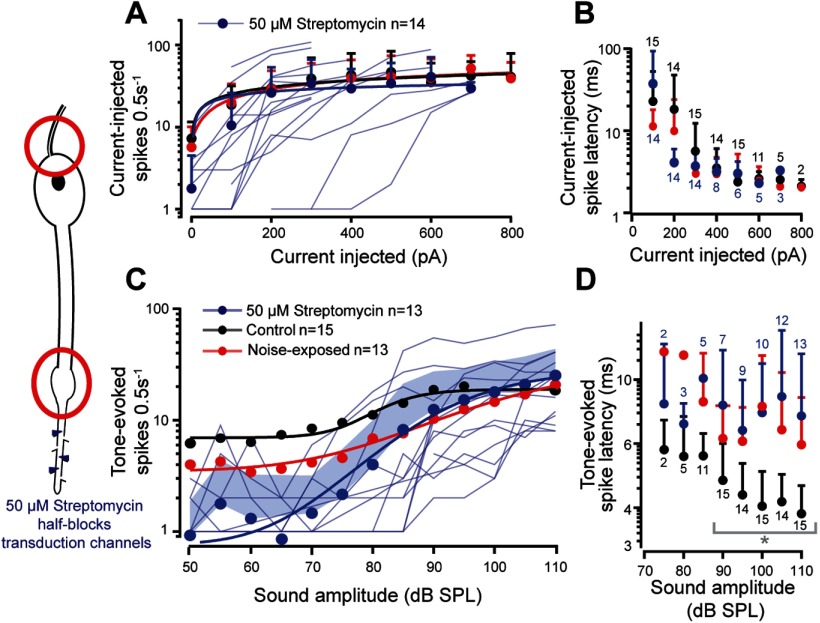
Axonal spikes at the axon hillock (red circle, top left) and dendritic spikes (red circle, bottom left) were measured in response to 50 μm streptomycin (blue triangles, left). ***A***, Number of current-injected spikes for noise-exposed (red), control (black), and streptomycin-perfused (blue) auditory neurons, fitted with a power law (solid lines) were not different between all three treatments. Means are plotted as circles, positive SD is plotted as error bars, current-elicited spikes from individual auditory neurons in the presence of 50 μm streptomycin are plotted as thin blue shaded lines (Control: *n* = 12, *N* = 9; noise-exposed: *n* = 11, *N* = 10; streptomycin perfused: *n* = 14, *N* = 10). ***B***, Latency to first current-injected spike for noise-exposed (red), control (black), and streptomycin perfused (blue) auditory neurons was not different between treatments (plotted on logarithmic axis for figure clarity). ***C***, Tone-evoked spikes for noise-exposed (red), controls (black), and streptomycin perfused auditory neurons fitted with a Boltzmann equation (streptomycin *R*^2^ = 0.990). Mean is plotted as circles, positive SD is plotted as shaded area and tone-evoked spike counts, from individual auditory neurons in the presence of 50 μm streptomycin, are plotted as thin blue lines. ***D***, Tone-evoked spike latency for noise-exposed (red), controls (black), and streptomycin perfused (blue) auditory neurons (plotted on logarithmic axis for figure clarity). Gray brackets with asterisk donate the recordings that were statistically tested (for justification, see Materials and Methods, Experimental design and statistical analysis).

We showed a decreased amplitude of spontaneous openings (discrete depolarizations) of the mechanotransduction ion channels (Fig. 3*E*) and decreased tone-evoked transduction current after noise exposure (Fig. 3*J*). To mimic this effect we used the known mechanotransduction ion channel blocker dihydrostreptomycin to half-block the mechanotransduction channels of control locusts not exposed to noise (Warren and Matheson, 2018). Blocking of the mechanotransduction ion channels had no effect on the ability to elicit spikes through current injection into the soma (Fig. 6*A*; LMEM: *t*_(39.38)_ = 0.087, *p* = 0.9313) or their latency (Fig. 6*B*; Control vs Noise-exposed, LMEM: *t*_(76.33)_ = −1.189, *p* = 0.237; Control vs Streptomycin, LMEM: *t*_(84.42)_ = 0.646, *p* = 0.520; Noise-exposed vs Streptomycin, LMEM: *t*_(84.5)_ = −1.838, *p* = 0.0696), thus mimicking auditory neurons from noise-exposed ears. The application of 50 μm streptomycin reduced the tone-evoked spikes compared with control (LMEM: *t*_(241.76)_ = −2.970, *p* = 0.0033; which was similar to noise-exposed auditory neurons only for higher SPLs >85 dB SPL; Fig. 6*C*). The spontaneous spike rate, which led to stark differences in the number of tone-evoked spikes at lower SPLs, was significantly lower in the presence of 50 μm streptomycin compared with control locust auditory neurons (Fig. 6*C*) and tended to be lower compared with noise-exposed auditory neurons. The latency of tone-evoked spikes was increased in the streptomycin treated auditory neurons, compared with controls (LM: *t*_(172)_ = 7.241, *p* < 0.0001), but remained similar to the noise-exposed locusts (Fig. 6*D*; LM: t_t(142)_ = 1.816, *p* = 0.0711).

### Transcriptome analysis reveals no change in expression of the mechanotransduction ion channel candidates

**Figure 7.**
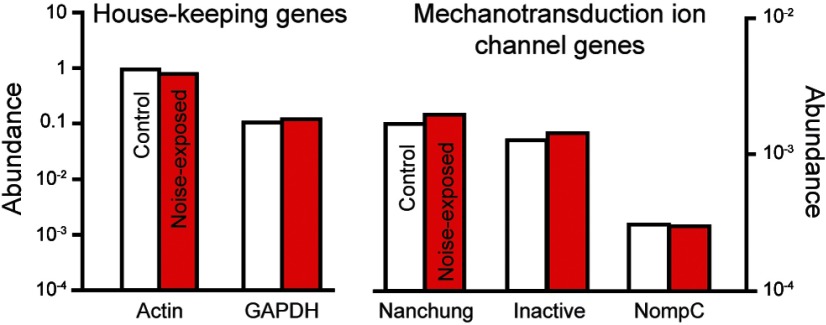
Abundance of RNA transcripts extracted from Müller's organs of control and noise-exposed locusts for house-keeping genes, *actin* and *GAPDH* and for the three genes that together code the two mechanotransduction ion channel candidates, *nanchung*, *inactve*, and *nompC*. Abundance was calculated by counting all matching reads to each gene's open reading frame in the complete, groomed transcriptomes, and then normalized by the reading frame length.

The reduction in the transduction current could be explained by a reduction in expression of the mechanotransduction ion channels. To measure expression of the putative transduction ion channels we extracted RNA from 320 Müller's organs from 160 control and noise-exposed locusts (2 ears per locust). We analyzed the RNA reads (sequenced by Beijing Genomics Institute) and quantified expression levels for the three genes that compose the two mechanotransduction ion channel candidates in insects: *nompC*, *nanchung*, and *inactive* (*nanchung* and *inactive toge*ther code a single Nanchung-inactive ion channel) and two housekeeping genes *actin* and *GAPDH*. We found no change in the expression level of any three of the genes that code the candidate mechanotransduction ion channels (Fig. 7).

## Discussion

Although most noise-induced hearing loss begins with the overstimulation of primary auditory receptors we lack a characterization of their electrical properties and electrical currents after acoustic overexposure for any animal ear. We used the physiologically accessible tympanal ear of the locust to quantitatively characterize changes in auditory neurons after noise exposure.

### Biomechanical responses of the tympanum

Sound-induced movements of human tympani, and the noises they produce (otoacoustic emissions), are routinely used to assay the health of the auditory receptors buried deep within the inner ear. Thus, the tympanum is not a simple passive receiver but can be influenced by the active force-producing mechanical properties of the primary auditory receptors themselves. Although first discovered in humans (Kemp, 1978), this principle extends to the ears of reptiles (Manley et al., 2001), amphibians (Long et al., 1996), birds (Taschenberger and Manley, 1997), and multiple insects (Göpfert and Robert, 2001, 2003; Mhatre and Robert, 2013). We found that the tympani of noise-exposed locust ears vibrate ∼10 times higher than controls across sound levels from 50 to 100 dB SPL. This drastic increase in compliance has three probable explanations. (1) Disruption of a possible active force-producing process intrinsic to the auditory neurons themselves (Möckel et al., 2007), (2) disruption of cytoskeletal components in the cells of Müller's organ, and (3) fatigue of a muscle that could act like the middle ear muscle of humans that contracts to protect the ear against loud damaging noise. Changes in the mechanics of the locust ear will, in turn, lead to changes in the ability of the auditory neurons to transduce these mechanical movements into action potentials. Thus, we next measured tone-evoked action potentials that are carried along the auditory nerve.

### *In vivo* electrophysiological responses of the auditory nerve

The health of an auditory system, its ability to transduce sound into electrical signals, has been assayed from the summated electrical potentials of the auditory nerve or auditory processing areas in the brain. After acoustic overstimulation there is an increase in the auditory threshold (Van Heusden and Smoorenburg, 1981; Pettigrew et al., 1984; Dolan and Mills, 1989; Ma et al., 1995; Sendowski et al., 2004; Telang et al., 2010; Pilati et al., 2012; Housley et al., 2013; Christie and Eberl, 2014; Coyat, et al., 2019) and a decrease in the sound-evoked compound action potential amplitude (Van Heusden and Smoorenburg, 1981; Pettigrew et al., 1984; Dolan and Mills, 1989; Wang et al., 1992; Sendowski et al., 2004; Christie and Eberl, 2014). In the locust we found, first, that the linear displacement of the tympanum is converted into a nonlinear sigmoidal electrical response of the tympanal nerve. This is presumably because of the nonlinear filter properties inherent in the mechanoelectrical gating of transduction ion channels (Hummel et al., 2016). In the auditory nerve we found, that the number of sound-elicited nerve potentials, was mildly decreased and their peak response, which represents spike synchrony, was strongly decreased after noise-exposure. The latency to first spike was significantly increased in noise-exposed locusts compared with controls, which reflects increased latencies measured in other noise-exposed auditory systems (Van Heusden and Smoorenburg, 1981; Pettigrew et al., 1984; Sendowski et al., 2004; Christie and Eberl, 2014). A likely cause of increased latencies after noise exposure is due to an increased auditory threshold. Likewise, a probable cause of a decrease in spike synchrony, or peak response, is a more variable latency. Is it not known what changes take place in the auditory receptors to decrease the synchrony of nerve responses and increase spike latency. To pinpoint noise-induced initial changes we performed whole-cell patch-clamp recordings from primary auditory neurons.

### Physiological basis of a decrease in the transduction current

The electrical properties of the auditory neurons such as their membrane resistance, resting membrane potential and capacitance were not affected by noise exposure, although the power of our analyses was limited by our sample size. Next, we analyzed changes in the spontaneous and sound-evoked openings of the transduction ion channels (Hill, 1983a; Warren and Matheson, 2018). There was a significant reduction in the magnitude of sound-evoked transduction current and an increase in the latency to elicit the transduction current. We tested whether potential noise-induced changes in both, the dendritic and axonal spike generating machinery resulted in fewer sound-evoked spikes but found no difference in the threshold, latency, or spike width between noise-exposed and control auditory neurons. The decreased tone-evoked spikes are presumably a direct consequence of the reduced transduction current. The decreased transduction current measured here have multiple explanations: (1) the mechanical attachment of the auditory neurons to the tympanum, and the sound-induced force delivered to them, has weakened; (2) the number of transduction channels is reduced; and (3) the electrochemical driving force for the ions passing through the transduction channels is decreased. Ciliated auditory receptors across animals function with common biophysical principles (Howard and Hudspeth, 1988; Albert et al., 2007), share striking genetic homology (Wang et al., 2002) and (most probably) evolved from the same ancestral auditory receptor (Fritzsch and Beisel, 2004). Thus, we address each of these three explanations in a comparative context with other auditory systems across the animal kingdom.

#### Explanation 1: a decrease in the sound-induced force

The transduction current in noise-exposed auditory neurons is reduced by one-half despite a 10-fold increase in sound-evoked tympanal displacements. The increased movement of the locust tympanum mirrors a likely increase in compliance of the human tympanum measured in soldiers exposed to impulse noise (Job et al., 2016). In the locust ear there must be a drastic decrease in mechanical coupling between the tympanum and the auditory neuron cilia where these sound-induced forces open transduction ion channels, otherwise we should expect an increase of the transduction current after noise exposure. Despite this, the maximal transduction current asymptotes for sound amplitudes approaching 110 dB SPL (Fig. 3*I*) suggesting that all the transduction channels are opened at these higher sound levels. All together it seems unlikely that a reduction in the sound-induced force is the main cause of the decreased transduction current.

#### Explanation 2: a decrease in the transduction channel number

The second explanation is particularly hard to unequivocally test because the protein/s that form/s the transduction channel have, controversially, not been identified in insects, or indeed any animal ear (although in mammals, it seems, they are getting close; Qiu and Müller, 2018). We showed no change in expression of the three genes that compose the two putative candidate mechanotransduction ion channels. Providing that either Nanchung-inactive or NompC are the transduction channel, the decrease in the transduction current is probably because of a decreased electrochemical potential in the receptor lymph cavity that bathes extracellular surface of the, still elusive, mechanotransduction ion channel.

#### Explanation 3: a decrease in the electrochemical driving force

We have shown no large reduction in the intracellular potential of the auditory neurons, despite a large decrease in the transduction current. Thus, if a decrease in electrochemical gradient causes a decreased transduction current, it must result in a decrease in the extracellular electrochemical potential maintained in a specialized receptor lymph cavity that bathes the external surface of the transduction channels. This could result from damage in the supporting cells that pump ions. Indeed, in the fruit fly, knocked down expression of the sodium pump (Na^+^/K^+^ ATPase) in the supporting scolopale cells that enclose the receptor lymph result in deafness and anatomic abnormalities in the receptor lymph space and sensitization to acoustic trauma (Roy et al., 2013; Christie and Eberl, 2014). A similar result is found in mammals where the supporting cells (in the stria vasculairs) are oxidatively damaged after noise exposure (Shi et al., 2015) and there is also a concurrent decrease in the endocochlear potential (Hirose and Liberman, 2003) and normal concentrations of potassium and calcium ions (Vassout, 1984; Ma et al., 1995; Li et al., 1997). It is presumably the ATP-hungry process of maintaining high electrochemical gradients, through transmembrane Na^+^/K^+^ pumps, which makes the supporting cells a vulnerable target of noise exposure across animal phyla. This reduction in the transduction current leads to less spontaneous and sound-evoked spikes, which reduces the metabolic demands for these processes. Thus, this change may, in fact, serve as a protective mechanism by sparing further metabolically demanding processes in auditory receptors.

## Conclusion

This study presented here is the first systematic assessment of changes in primary auditory receptors in any animal after noise exposure. The electrophysiological properties, dendritic and axonal spike properties and expression of putative mechanotransduction ion channels of the auditory neurons were unchanged, demonstrating the resilience of auditory neurons in the face of a demanding acoustic insult. These findings mirror recent work on mammals that show an increase in hearing thresholds but without any loss of their primary auditory receptors (Kujawa and Liberman, 2009; Lin et al., 2011). Thus, a shared feature of auditory receptors, be it hair cells of mammals or primary auditory neurons of insects, is the incredible amount of resilience, possibly reflecting the evolutionary importance and survival value of maintaining sensitive hearing.
